# Methamphetamine Exposure in Adolescent Impairs Memory of Mice in Adulthood Accompanied by Changes in Neuroplasticity in the Dorsal Hippocampus

**DOI:** 10.3389/fncel.2022.892757

**Published:** 2022-05-17

**Authors:** Min Liang, Li Zhu, Rui Wang, Hang Su, Dongliang Ma, Hongyan Wang, Teng Chen

**Affiliations:** ^1^College of Forensic Medicine, Xi’an Jiaotong University Health Science Center, Xi’an, China; ^2^The Key Laboratory of Health Ministry for Forensic Science, Xi’an Jiaotong University, Xi’an, China; ^3^Programme in Neuroscience and Behavioral Disorders, Duke-NUS Medical School, Singapore, Singapore; ^4^Department of Physiology, Yong Loo Lin School of Medicine, National University of Singapore, Singapore, Singapore

**Keywords:** methamphetamine, adolescent, hippocampus, memory, neuroplasticity

## Abstract

Methamphetamine (METH) has been shown to alter learning and memory by affecting the neuroplasticity of the dorsal hippocampus, a key structure that undergoes extensive remodeling during adolescence. In this study, we investigated whether mid-to-late adolescent exposure to METH leads to long-lasting memory impairment. To do this, adolescents (35–48 postnatal days) were exposed to different doses of METH for 14 days and then evaluated by the Morris water maze (MWM), new object recognition test (NORT), and the Y-maze, to investigate the learning and memory abilities of mice in their adolescence and adulthood, respectively. We also detected the mRNA levels of genes associated with neuroplasticity in the dorsal hippocampus. The synaptic ultrastructure and the number of neurons and astrocytes in the dorsal hippocampus were also determined by transmission electron microscopy (TEM) and immunofluorescence (IF). Exposure to METH in mid-to-late adolescence impaired spatial memory retrieval ability and the long-term recognition memory of mice in their adulthood, but not in their adolescence. Of note, the impairment of memory capacity in adulthood was accompanied by molecular and structural changes in synapses in the dorsal hippocampus. Our results indicate that mice exposed to METH in mid-to-late adolescence have impaired memory ability in their adulthood; this may be the result of abnormal changes in the structural plasticity of the dorsal hippocampus; the causal relationship between changes in synaptic structural plasticity and memory impairment needs to be further confirmed. In summary, our study provides evidence for the detrimental consequences of adolescent addiction and the prevention of adolescent drug abuse.

## Introduction

Methamphetamine (METH) is a widely abused psychostimulant with strong neurotoxicity and obvious mental symptoms (Liu et al., 2021). Unfortunately, most METH abusers begin their exposure as teenagers. According to the recent data, half of all first-time drug abusers in China were adolescents and young people under the age of 25 years (Zhuang and Chen, 2016); this compares to a mean age of 18.9 years in the United States (Luikinga et al., 2018). The dominant feature of the adolescent brain is that synapses undergo extensive strengthening and elimination (Zeid et al., 2018). Furthermore, the adolescent brain is highly sensitive to stimuli from the internal and external environments (Smiley et al., 2021). Unbalanced development of the dopaminergic and GABAergic systems during adolescence has been shown to lead to an increased risk of motivation and decision-making among adolescents which may increase the use of psychoactive substances (Luna et al., 2015). This leads to abnormal structural changes in the brains of users with a history of adolescent abuse, making them more prone to substance abuse, emotional changes, and cognitive impairment in adulthood than normal adults (Levine et al., 2017; D’Amico et al., 2021). The rate of substance abuse in mid-to-late adolescence is significantly higher in adolescents who abuse drugs than those in early adolescence (Marmorstein et al., 2010; McCabe et al., 2019); this may be the result of an increase in the risk factors related to social context (Staff et al., 2010; D’Amico et al., 2021). However, most studies on adolescent METH exposure have focused on early adolescence (Joca et al., 2014; Buck et al., 2017). Therefore, in this study, we aimed to determine the effects of long-term exposure to METH during mid-to-late adolescence on learning and memory.

The long-term abuse of METH may ultimately affect learning and memory by altering the neuroplasticity of related brain regions such as the dorsal hippocampus. Studies have shown that the administration of METH can reduce the expression of synaptic molecules in the hippocampus, such as *Arc*, *c-fos*, and *Bdnf*, which are immediate early genes and can prompt rapid and transient synaptic activation responses while initiating signaling cascades to modulate neuronal plasticity (Minatohara et al., 2015; Hogan et al., 2020). Meanwhile, METH exposure can damage neurons and lead to a decline in the learning and memory abilities of adult mice (Ru et al., 2021; Veschsanit et al., 2021). In addition, METH increases glutamate levels in dorsal hippocampus synapses by reducing glutamate clearance in astrocytes and reduces postsynaptic neuronal activity, ultimately impairing spatial memory in adult mice (Robin et al., 2018; Hosseini et al., 2020). Moreover, changes in the ratio of gray to white matter in the brains of METH users in population studies have been associated with cognitive impairment (Lyoo et al., 2015). Collectively, these studies indicate that changes in neuroplasticity may be the main neural basis for the effects of drug exposure on learning and memory. However, whether exposure to METH in mid-to-late adolescence impairs learning and memory abilities in adulthood by affecting the neuroplasticity of the dorsal hippocampus remains unclear.

In this study, we investigated the effects of long-term exposure to METH during mid-to-late adolescence on learning and memory and determined the changes in neuroplasticity in the dorsal hippocampus. To do this, adolescent mice were exposed to METH for 14 days. We then investigated the effects of METH exposure on learning and memory during adolescence and adulthood by performing the Morris water maze (MWM), new object recognition test (NORT), and the Y-maze test (Antunes and Biala, 2012; Cao et al., 2018; Yan et al., 2019). The expression of plasticity-related molecules (*Arc*, *c-fos*, and *Bdnf*) in dorsal hippocampus was detected by real-time quantitative polymerase chain reaction (RT-qPCR). Furthermore, transmission electron microscopy (TEM) and immunofluorescence (IF) were used to identify the changes in synaptic plasticity, including synaptic ultrastructure and the number of neurons and astrocytes in different subregions of the dorsal hippocampus.

## Materials and Methods

### Animals

During our studies, we used 4-week-old adolescent male C57BL/6J mice weighing 15–18 g (Beijing Vital River Laboratory Animal Technology, Beijing, China). Mice were randomly divided into groups of four individuals per cage under controlled conditions (12-h light/dark cycle from 7:00 to 19:00, 50 ± 10% humidity, and 23 ± 2°C temperature control) with food and water *ad libitum*. Mice were accustomed to this experimental environment for 7 days and allowed to adapt prior to each individual experiment. There were 36 mice in the saline (Sal) group and 36 mice in the METH group. Animal procedures were conducted in accordance with the United Kingdom Animals (Scientific Procedures) Act and Institutional Animal Care Committee at Xi’an Jiaotong University.

### Drugs

Methamphetamine hydrochloride (National Institute for the Control of Pharmaceutical and Biological Products, Beijing, China) was dissolved in 0.9% physiological saline to a concentration of 0.2 mg/ml and injected intraperitoneally at a volume of 10 ml/kg (i.p.). All mice were consecutively exposed to corresponding concentrations of METH (1, 2 mg/kg) or saline one time a day from 9:00 a.m. to 11:00 a.m. for 14 days after adaptation (35–48 postnatal days). The rationale for the dosing regimen was based on a previous study that demonstrated that the treatment of mice with METH for 14 days led to a clear impairment in recognition memory (North et al., 2013).

### Morris Water Maze

The Morris Water Maze (MWM) protocol was based on a previous study with some modifications (Cao et al., 2018). In brief, the surface of the water was artificially divided into four quadrants, each with an entry point. Signs of the same size and color, but in different shapes, were pasted in the middle of the water tank wall in each quadrant. During the spatial learning phase, a plexiglass platform with a diameter of 10 cm was hidden 1–1.5 cm below the water surface in the second quadrant; this platform was subsequently removed until the probe test phase. A camera was placed above the tank and was used to automatically track each animal’s performance with a video-computerized tracking system (SMART, Panlab SL, Barcelona, Spain).

The MWM was performed during adolescence or adulthood; there were eight mice in the METH group or Sal group in different periods (adolescence, adulthood) and at different doses (1, 2 mg/kg), respectively. The experiment was divided into a spatial learning phase (four trials a day for 4 or 5 days) and a probe test phase (24 h after the last trial). During the learning phase, the mice were permitted to acclimatize to the environment for 1 day before the learning phase; they were then placed randomly in the water facing the tank wall at one of four fixed entry points. If the mice climbed onto the platform within 60 s, they were allowed to stay there for 10 s and the latency was recorded; if not, the mice were manually placed on the platform for 15 s and the latency was recorded as 60 s. After each trail, the mice were wiped with a towel and placed in their home cage for at least 10–15 min until the next training session. The mean latency and speed in four trails per day were set as the key indices for the learning ability of the mice. During the probe test phase, the mice were placed in water from the point farthest away from the platform and recorded for 60 s without the platform. During this phase, the spatial memory retention ability was represented by the number of platform site crossings, the time in the target quadrant, and the time in the target.

### New Object Recognition Test

The procedure used for the New Object Recognition Test (NORT) was described previously (Antunes and Biala, 2012). The 4-day experiment was carried out in adulthood after exposure to 2 mg/kg of METH during adolescence (*n* = 8). The mice were adapted to the empty box for 10 min a day for the first 2 days of the experiment. During the familiarization phase on day 3, two identical objects were placed at an equal distance from the box, and the mice were allowed to explore freely for 5 min. Each mouse was required to perform three training sessions at 15-min intervals. A short-term memory test (test 1) was performed 3 h after the last training session, in which one object (A) was replaced by another object (B) of the same size but a different shape. The long-term memory test (test 2) was administered 24 h after the last training session and was the same as the short-term memory test, except that object (B) was replaced by another new object (C); the time was recorded for 5 min. We also determined the recognition index (%). The top of the box was recorded by the camera and finally analyzed by software (SMART, Panlab SL, Barcelona, Spain).

### Y-Maze Spontaneous Alternation Test

The Y-maze spontaneous alternation test was carried out as described previously (Yan et al., 2019). The experiment was conducted 1 week after the NORT (*n* = 8). During this experiment, each mouse was placed at the central point and allowed to explore freely for 5 min; we recorded the order in which the animals entered each arm. Alternation was defined as continuous entry into three different arms, such as (A, B, C or C, A, B). Next, we calculated the alternation triplet (%), as follows: Alternation triplet (%) = actual alternative/maximum alternative × 100%. Mice with less than eight total arm entries were excluded. The behavioral indicators for each mouse were recorded by specific software (SMART, Panlab SL, Barcelona, Spain).

### Quantitative Real-Time PCR

After the behavioral experiments, the mice were sacrificed (*n* = 6) and their brains were quickly removed. Next, we prepared tissue sections from the dorsal hippocampus (Franklin and Paxinos, 2001) using a cryostat; these sections were stored at –80°C until treatment. Then, total RNA was isolated using an E.Z.N.A. DNA/RNA/Protein Kit (Omega Bio-Tek, Norcross, GA, United States). The concentration and mass of the extracted RNAs were determined by a Nano Drop spectrophotometer (Thermo Scientific, Waltham, MA, United States). Next, 500 ng of total RNA was reverse transcribed-into 10 μl of cDNAs with a PrimeScript™ RT Master Mix (Takara Biomedical Technology, Beijing, China) at 37°C for 15 min, 85°C for 5 s, and 4°C for 5 min. Then, we determined the relative expression levels of *Arc*, *c-fos*, and *Bdnf* mRNA in dorsal hippocampus of mice. qPCR was performed with SYBR Premix Ex Taq II (Takara Biomedical Technology, Beijing, China) and a Bio-Rad iQ5 detection instrument (Bio-Rad, Hercules, CA, United States) under the following conditions: 95°C for 30 s, followed by 40 cycles of 95°C for 10 s, 60°C for 30 s, and 72°C for 30 s. After the completion of the reaction, specificity was verified by melting curve analysis. The relative mRNA values were normalized to the control values of the *Gapdh* gene and calculated using the 2^–△△*Ct*^ method (Livak and Schmittgen, 2001). The sequences of the forward (F) and reverse (R) primers are shown in Table 1.

**TABLE 1:** Primers used for quantitative polymerase chain reaction (qPCR).

Gene	T_*m*_ (°C)	Forward (5′–3′)	Reverse (5′–3′)
*Gapdh*	60	TGTGTCCGTCGTGGATCTGA	TTGCTGTTGAAGTCGCAGGAG
*Arc*	60	GCCAAACCCAATGTGATCCTG	CTGCTTGGACACTTCGGTCAAC
*c-fos*	60	CGACTCCTTCTCCAGCAT	TCACCGTGGGGATAAAGTTG
*Bdnf*	60	CTGAGCGTGTGTGACAGT ATTAGC	GTAGTTCGGCATTGCGA GTTCC

### Transmission Electron Microscope Analysis

To prepare tissue for Transmission Electron Microscope (TEM), mice (*n* = 3) were exposed to 2 mg/kg of METH and then anesthetized after completing the final behavioral experiment in adulthood. The brain tissue was fixed by left ventricular vascular perfusion; we injected the following in sequence: 0.9% physiological saline, 0.1 M phosphate buffer (PB), and a mixed solution of 4% paraformaldehyde and 0.25% glutaraldehyde (pH 7.4). Then, the brain was removed and the bilateral dorsal hippocampus were extracted and dissected into tissue blocks that were approximately 1 mm^3^ in size. These small tissue blocks were then fixed in 0.1 M PB containing 4% paraformaldehyde and 2.5% glutaraldehyde (pH 7.4) at 4°C for at least 2 h. Next, the samples were immersed in 0.1 M PB for more than 30 min, followed by fixation in a fresh 1% solution of osmium tetroxide for 120 min and immersed in 0.1 M PB for 10 min. Next, the tissues were dehydrated in different concentrations of ethanol and then embedded in Epon-Araldite resin. Next, the brain tissue was cut into 1-μm-semi-thin sections, stained with methylene blue, and then cut into 50-nm-ultra-thin sections. A total of three ultra-thin sections of each mouse were randomly selected for photography, and 3–5 images of each ultra-thin section were taken for statistical analysis. Images were acquired by an electron microscope (H-7650, Hitachi Limited, Tokyo, Japan), so that we could analyze the number of synapses (magnification 20,000×; 30 photographs) and the synaptic ultrastructure (magnification 40,000×; 38 photographs). For analysis, synapses needed to contain three or more synaptic vesicles in their presynaptic elements and obvious dense layers in the postsynaptic elements (Wang et al., 2020). Image Pro Plus 6.0 (Media Cybernetics, Rockville, MD, United States) was used to measure the thickness of postsynaptic density (PSD) at the thickest part, the width of the synaptic cleft, the length of the presynaptic active zone, and the total number of synapses. All analyses were performed in a blinded manner.

### Immunofluorometric Assay

Mice (*n* = 3) were deeply anesthetized and then fixed by brain perfusion with 0.9% physiological saline and 4% paraformaldehyde *via* the left ventricular vessels. Then, the brain tissue was fixed with 4% paraformaldehyde at room temperature for 4 h and dehydrated with 30% sucrose solution. When completely dehydrated, the tissues were embedded with optimal cutting temperature compound and frozen in liquid nitrogen. Coronal sections (20 μm) at the hippocampal level were prepared using a freezing microtome (Leica CM1850, LEICA, Wetzlar, Germany). Subsequently, sections were blocked with 5% bovine serum albumin at 37°C for 60 min. Subsequently, brain tissue sections were incubated with chicken-anti GFAP (1:200; Abcam Cat# ab134436, RRID:AB_2818977) and rabbit-anti NeuN (1:100; Abcam Cat# ab177487, RRID:AB_2532109) specific primary antibody mixed solution at 4°C for 24–48 h. In the following morning, the sections were washed with phosphate buffer saline (PBS) and incubated with goat anti-chicken Alexa fluor 488 (1:500; Abcam Cat# ab150173, RRID:AB_2827653) and goat anti-rabbit coralite594 conjugate (1:500; Proteintech Cat# SA00013-4, RRID:AB_2810984) for 90 min at room temperature, respectively. Finally, after 5 min of incubation with 4′,6-diamidino-2-phenylindole (DAPI), stained sections were examined under a fluorescence microscope (Carl Zeiss, Axio scope A1, Germany); 10–15 regions were randomly selected from each group. The positive cell density was determined and the NeuN/GFAP ratio was calculated. All stained sections were analyzed by observers who were unaware of the experimental cohort.

### Statistical Analysis

Statistical analyses were performed using SPSS 18.0 (IBM, Armonk, NY, United States) or Prism 8.0 (Blue Prism, London, United Kingdom). Data collection and analysis were not performed blinded to the experimental conditions. The mice were randomly assigned into study groups. All data are presented as the mean ± standard error of the mean (SEM). The two-way repeated measures analysis of variance (ANOVA) was used to analyze the latency to platform in the MWM, and a post hoc multiple comparison (Bonferroni test) was used to analyze the differences between different days. Other data were analyzed by the Student’s *t*-test according to the homogeneity of variance. *p*-Values < 0.05 were considered statistically significant.

## Results

### Exposure to Methamphetamine During Adolescence Did Not Affect the Learning and Memory Abilities of Mice in Adolescence

**FIGURE 1:**
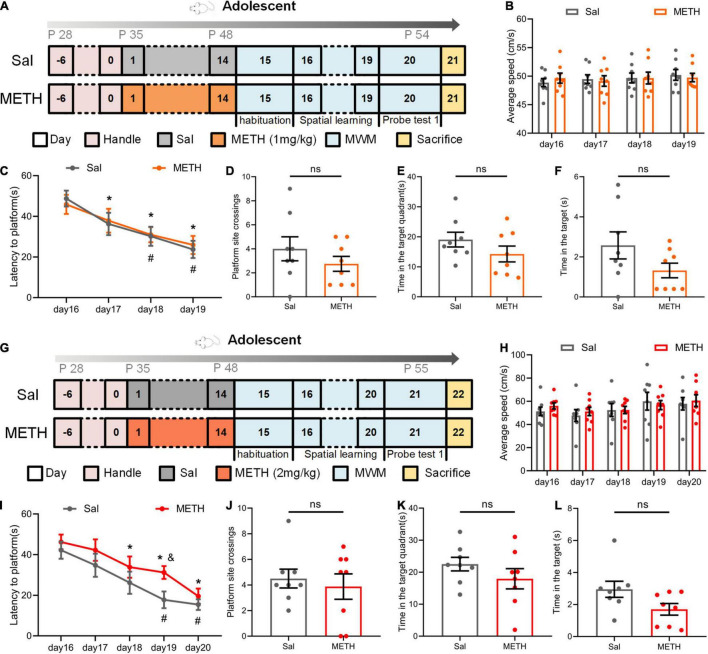
Exposure to Methamphetamine (METH) during adolescence did not impair the memory ability of mice during this period of development. **(A–F)** The results of the Morris water maze (MWM) in adolescent mice after exposure to 1 mg/kg of METH. **(A)** Experimental protocol. **(B)** Mean swimming speed in the spatial learning phase. **(C)** Latency to the platform (day: F_(3,42)_=10.505, *p* < 0.001; group: F_(1,14)_ = 0.062, *p* > 0.05; day × group: F_(3,42)_ = 0.147, *p* > 0.05). *p < 0.05 compared with day 16 in the saline group (Sal). #*P* < 0.05 compared with day 16 in the METH group (METH). **(D)** Platform site crossings. **(E)** Time in the target quadrant. **(F)** Time in the target. **(G–L)** The results of the MWM in adolescent mice after exposure to 2 mg/kg of METH. **(G)** Experimental protocol. **(H)** Mean swimming speed in the spatial learning phase. **(I)** Latency to the platform [day: F_(4, 56)_ = 16.564, *p* < 0.001; group: F_(1, 14)_ = 3.188, *p* > 0.05; day × group: F_(4, 56)_ = 0.529, *p* > 0.05]. *p < 0.05 compared with day 16 in the Sal group. ^#^*p* < 0.05 compared with day 16 in the METH group. ^&^*p* < 0.05 METH group compared with the Sal group. **(J)** Platform site crossings. **(K)** Time in the target quadrant. **(L)** Time in the target. Data are presented as mean ± SEM, *n* = 8.

To investigate the effect of METH on learning and memory in adolescent mice (49–54 postnatal days), we first exposed mice to 1 mg/kg of METH for 14 days (35–48 postnatal days). MWM was performed immediately after drug exposure to identify the changes in the learning and memory abilities of mice (Figure 1A). During the spatial learning phase, as the number of training days increased, the latency of the mice to the platform shortened; however, there was no significant difference between the two groups with this respect (Figure 1C, when compared to day 16 of the same group, *p* < 0.05). We also tested the swimming speed to exclude potential interference effects; there was no significant difference between the groups (Figure 1B). In addition, probe test 1 showed that there was no significant difference between the groups in terms of platform site crossings (Figure 1D), the time in the target quadrant (Figure 1E), and the time in the target (Figure 1F).

Next, we changed the dose of METH. Adolescent mice were exposed to 2 mg/kg of METH for 14 days, and the MWM was also performed in adolescence (Figure 1G). The results were very similar to those seen with the 1 mg/kg treatment (Figures 1H–L). During the spatial learning process, the latency to the platform in all groups of mice decreased as the number of training days increased, although the drug had no significant effect (Figure 1I, compared with day 16 of the same group, *p* < 0.05). We also found that speed had no significant effect on latency (Figure 1H). In probe test 1, there was no significant difference between the Sal group and the METH group in terms of platform site crossings (Figure 1J), the time in the target quadrant (Figure 1K), and the time in the target (Figure 1L). These results showed that long-term exposure to METH during adolescence did not affect the learning and memory abilities of mice in their adolescence.

### Exposure to Methamphetamine During Adolescence Impaired Spatial Memory of Mice in Adulthood

**FIGURE 2:**
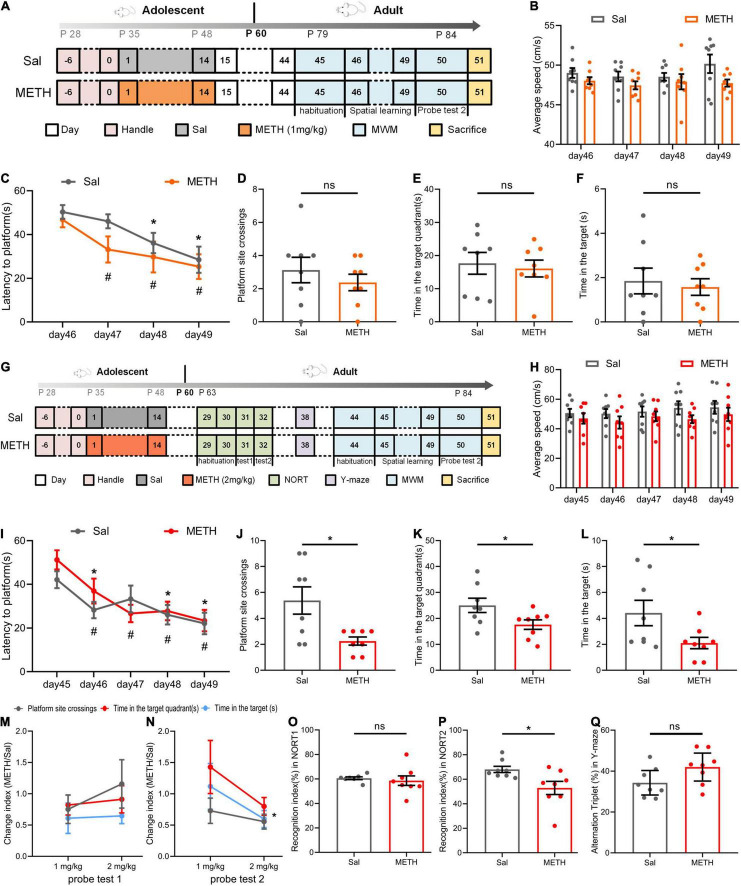
Exposure to METH during adolescence impaired the memory ability of mice in adulthood. **(A–F)** The results of the MWM in adulthood after exposure to 1 mg/kg of METH during adolescence. **(A)** Experimental protocol. **(B)** Mean swimming speed in the spatial learning phase. **(C)** Latency to the platform [day: F_(3,42)_=13.275, *p* < 0.001; group: F_(1,14)_ = 1.307, *p* > 0.05; day × group: F_(3,42)_ = 0.780, *p* > 0.05]. *p < 0.05 compared with day 46 in the Sal group. ^#^*p* < 0.05 compared with day 46 in the METH group. **(D)** Platform site crossings. **(E)** Time in the target quadrant. **(F)** Time in the target. **(G–L)** The results of the MWM in adulthood after exposure to 2 mg/kg of METH during adolescence. **(G)** Experimental protocol. **(H)** Mean swimming speed in the spatial learning phase. **(I)** Latency to the platform [day: F_(4, 56)_ = 10.184, *p* < 0.001; group: F_(1, 14)_ = 0.447, *p* > 0.05; day × group: F_(4, 56)_ = 1.280, *p* > 0.05]. *p < 0.05 compared with day 45 in the Sal group. ^#^*p* < 0.05 compared with day 45 in the METH group. Platform site crossings **(J)**, time in the target quadrant **(K)**, and time in the target **(L)** in probe test 2 were decreased. *p < 0.05 compared with the Sal group. Memory ability indices in probe test 1 **(M)** and probe test 2 **(N)**. *p < 0.05 compared with the corresponding group. **(O)** Short-term memory recognition index in the new object recognition test (NORT). **(P)** Long-term memory recognition index was decreased in the NORT. *p < 0.05 compared with the Sal group. **(Q)** Alternation triplet in the Y-maze. Data are presented as mean ± SEM, *n* = 8.

Next, we investigated whether drug exposure in adolescence would cause changes in learning and memory abilities at adulthood (63–84 postnatal days). Mice were exposed to 14 days of METH (1 or 2 mg/kg). The MWM experiment was performed in adulthood to verify whether the learning and memory abilities had changed. As shown in Figures 2A–F, during the phase of spatial learning, all mice learned to find the platform faster as training continued (Figure 2C, when compared to day 46 of the same group, *p* < 0.05). Swimming speed had no effect on latency to the platform (Figure 2B). There were no significant differences arising from probe test 2 in terms of platform site crossings (Figure 2D), the time in the target quadrant (Figure 2E), and the time in the target (Figure 2F).

In contrast, exposure to 2 mg/kg of METH during adolescence impaired spatial memory retention but not acquisition in their adulthood. As shown in Figures 2G–L, during the training stage of the MWM (Figure 2I), the latency of mice climbing the platform was not affected by the drug after 5 days of training when the groups were compared (Figure 2I, when compared to day 45 of the same group, *p* < 0.05). Mean swimming speed did not have a significant effect on latency to the platform (Figure 2H). In probe test 2, the mice exposed to METH during adolescence underwent fewer platform site crossings [Figure 2J, t_(14)_=2.849, *p* < 0.05], less time in the target quadrant [Figure 2K, t_(14)_=2.233, *p* < 0.05], and less time in the target [Figure 2L, t_(14)_=2.158, *p* < 0.05]. We compared the mean METH/Sal ratio between different doses of exposure in probe test 1 (Figure 2M) and probe test 2 (Figure 2N), including platform site crossings, the time in the target quadrant, and the time in the target. This allowed us to investigate the dose effects on changes in spatial memory in adolescence (probe test 1) and adulthood (probe test 2). The smaller the index, the more serious damage was revealed. As expected, the damage caused by the 2 mg/kg dose was more serious than that caused by the 1 mg/kg dose in adulthood [t_(4)_=3.161, *p* < 0.05].

In addition, we conducted the NORT and Y-maze to investigate the cognitive ability of mice during adulthood (Figure 2G). The NORT showed that the short-term memory recognition ability of mice was not significantly affected (Figure 2O). However, during the long-term memory recognition stage (Figure 2P), the recognition index of the METH group was significantly lower than that of the Sal group [t_(14)_=2.547, *p* < 0.05]. The Y-maze was performed 7 days after the end of the NORT and revealed no change in the number of spontaneous alternations (Figure 2Q). Collectively, these results suggested that adolescent mice exposed to 2 mg/kg METH suffered from impaired spatial memory and long-term recognition memory in their adulthood.

### Exposure to Methamphetamine During Adolescence Resulted in the Reduced Expression of Genes Related to Dorsal Hippocampal Neuroplasticity in Adulthood

**FIGURE 3:**
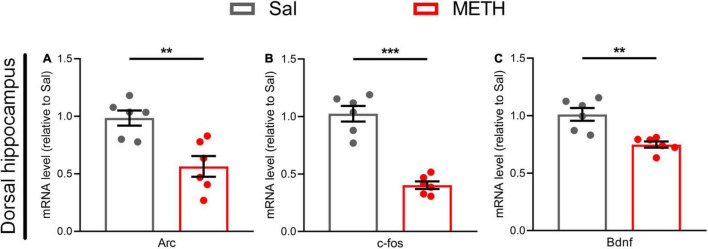
Exposure to METH during adolescence reduced the expression of genes related to synaptic plasticity in the dorsal hippocampus of mice in adulthood. The expression of genes related to neural plasticity in the dorsal hippocampus **(A–C)** in adulthood, including **(A)** *Arc*, **(B)** *c-fos*, and **(C)** *Bdnf*. Student’s *t*-test: **p < 0.01, ***p < 0.001 compared with the Sal group. Data are presented as mean ± SEM, *n* = 6.

To explore the neural plasticity mechanism of memory impairment in adulthood, we first examined the changes in the mRNA expression of genes related to neuroplasticity, including *Arc*, *c-fos*, and *Bdnf* in the dorsal hippocampus of mice exposed to METH (2 mg/kg) during adolescence. The expression levels of *Arc* [Figure 3A, t_(10)_=3.776, *p* < 0.01], *c-fos* [Figure 3B, t_(10)_=8.220, *p* < 0.001], and *Bdnf* [Figure 3C, t_(10)_=4.267, *p* < 0.01] in the METH group were significantly reduced when compared to controls.

### Exposure to Methamphetamine During Adolescence Resulted in Significant Alterations in the Ultrastructure of Synapses in the Dorsal Hippocampus During Adulthood

**FIGURE 4:**
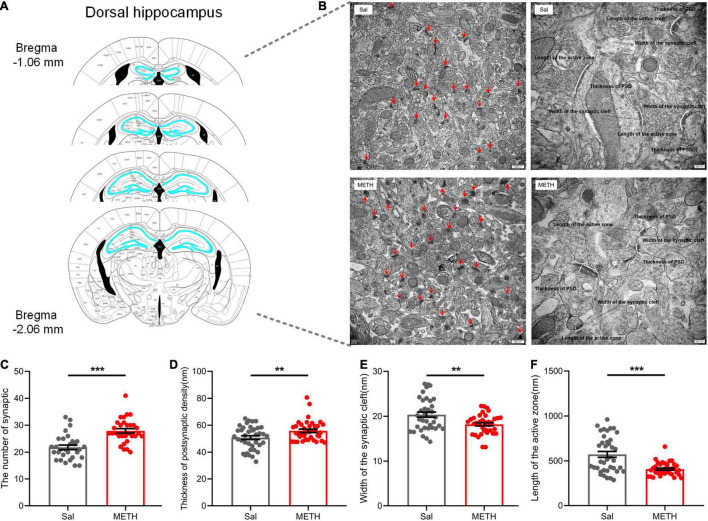
Exposure to METH during adolescence changed the synaptic ultrastructure in the dorsal hippocampus of mice in adulthood. **(A)** Bilateral dorsal hippocampus images of experimental mice. **(B)** Representative electron micrographs. Images on the left show representative images of the number of synapses (20,000×, scale bars = 500 nm); the red arrow points to a single synapse. Images on the right are representative images of the synaptic ultrastructure (40,000×, scale bars = 200 nm). The length of the segment shown by the white line represents the thickness of the PSD, the width of synaptic cleft, and the length of the presynaptic active zone, respectively. Histograms show the increase in synapse density **(C)** and the thickness of the PSD at its thickest part **(D)**, the reduction in width of the synaptic cleft **(E),** and length of the active zone **(F)**. **p < 0.01, ***p < 0.001 compared with the Sal group. Data are presented as mean ± SEM, *n* = 3.

We then investigated the changes in the ultrastructure of synapses in the dorsal hippocampus (Figure 4A) of adult mice that had been exposed to METH during their adolescence (2 mg/kg). Using TEM, we observed the number of synapses (the left side of Figure 4B, 20,000×) and the ultrastructure of the synapses (the right side of Figure 4B, 40,000×), including the thickness of the PSD, synaptic cleft width, and the length of the presynaptic activity zone. The statistical results are shown in Figures 4C–F; compared with the Sal group, the number of synapses [Figure 4C, t_(58)_=5.135, *p* < 0.001] and the thickness of the PSD [Figure 4D, t_(74)_=2.72, *p* < 0.01] in the METH group were significantly higher, whereas the width of the synaptic cleft was significantly reduced in the METH group [Figure 4E, t_(74)_=3.176, *p* < 0.01]. In addition, the length of the presynaptic activity zone was also reduced significantly in the METH group when compared to the Sal group [Figure 4F, t_(74)_=4.751, *p* < 0.001]. These results indicated that exposure to METH during adolescence changed synaptic ultrastructure in the dorsal hippocampus during adulthood; this may be the structural basis for spatial memory impairment.

### Exposure to Methamphetamine During Adolescence Resulted in a Significant Reduction in the Number of Neurons in the CA Region of the Dorsal Hippocampus of Mice in Their Adulthood

**FIGURE 5:**
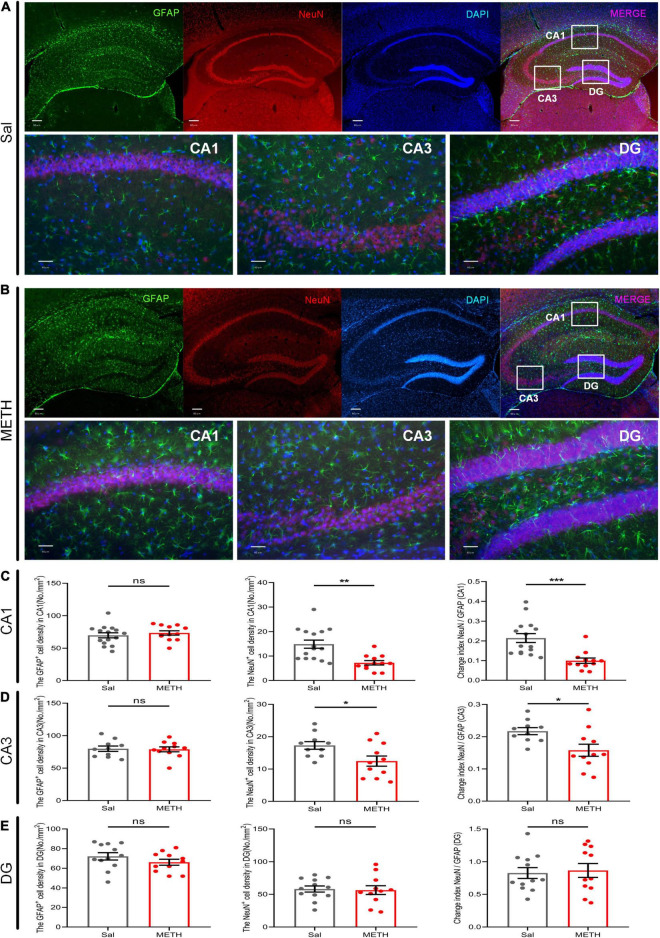
Exposure to METH during adolescence reduced the number of neurons in the dorsal CA region of mice in adulthood. Fluorescence representative images of the Sal group **(A)** and the METH group **(B)**. The upper part of the figure shows the localization of the CA1, CA3, and DG regions in the dorsal hippocampus under low magnification. Scale bar = 80 μm. The lower part of the figure shows the count image for each partition. Scale bar = 40 μm. Neuronal marker (NeuN, red) and astrocyte marker (GFAP, green). Nuclei (DAPI, blue). Panels **(C–E)** represent the density of GFAP-positive cells, NeuN-positive cells, and the ratio of NeuN to GFAP in the CA1, CA3, and DG, respectively. *p < 0.05, **p < 0.01, ***p < 0.001 compared with the Sal group. Data are presented as mean ± SEM, *n* = 3.

Structural plasticity includes not only synaptic changes but also the number of neurons and glial cells (Allen and Eroglu, 2017). Astrocytes are the most abundant glial cells in the central nervous system and have been repeatedly shown to be involved in neuroplasticity; many studies have reported that astrocytes are vital for learning and memory (Allen and Eroglu, 2017; Robin et al., 2018). Therefore, we labeled GFAP and NeuN to detect the changes in the number of astrocytes and neurons in the CA and dentate gyrus (DG) regions of the dorsal hippocampus in adult mice that had been exposed to METH during their adolescence (2 mg/kg) (Figure 5). There was no significant difference in terms of cell density of astrocytes in the CA1 region (Figures 5A,B) when compared between the METH group and the Sal group (Figure 5C); However, we identified a significant reduction in the cell density of neurons [Figure 5C, t_(21.175)_=4.025, *p* < 0.01], and the ratio of NeuN to GFAP [Figure 5C, t_(25)_=4.078, *p* < 0.001] in the METH group. Similarly, in the CA3 region (Figures 5A,B), there was no significant difference in the number of GFAP-positive cells in the METH group when compared to the Sal group (Figure 5D); however, the density of NeuN-positive cells was significantly lower [Figure 5D, t_(19)_=2.427, *p* < 0.05]. The ratio of NeuN to GFAP was also significantly reduced in the METH group [Figure 5D, t_(19)_=2.67, *p* < 0.05]. Surprisingly, there was no change in the DG region (Figure 5E). In summary, exposure to METH during adolescence can reduce the number of neurons in the CA region in adulthood.

## Discussion

The main findings of this study were 2-fold: (1) exposure to METH during mid-to-late adolescence impaired the memory ability of mice in their adulthood; and (2) the impairment of memory capacity in adulthood is accompanied by molecular and structural changes in the synapses of the dorsal hippocampus.

In our study, we found that exposure to 1 mg/kg or 2 mg/kg of METH during mid-to-late adolescence did not affect the cognitive level of mice at that time, whereas exposure to 2 mg/kg of METH during adolescence resulted in memory impairment in their adulthood. These findings are similar to those published previously which demonstrated cognitive impairment in mice caused by METH exposure during adolescence (Joca et al., 2014; Buck et al., 2017; Yan et al., 2019). One of the possible mechanisms by which METH exposure during adolescence leads to delayed cognitive impairment in adulthood is that METH disrupts the normal brain maturation during late adolescence, thus leading to the gradual development of behavioral deficits and pathological changes in synaptic structure (Grochecki et al., 2021). Previous studies have shown that the effect of continuous METH exposure may prevent learning and memory damages in mice during adolescence (Joca et al., 2014; Buck et al., 2017). This short-term protective mechanism may be due to the negative feedback effect of METH on the activation of the hypothalamic–pituitary–adrenal (HPA) axis, thus weakening the damaging effects of drugs; however, long-term activation of the HPA axis may lead to adverse effects (Acevedo et al., 2008; Hueston et al., 2017). As a key hub of the HPA axis, the paraventricular nucleus of the hypothalamus has been shown to be extensively involved in drug addiction over the recent years (Zhou and Zhu, 2019). Interestingly, the dorsal hippocampus, which is closely associated with cognitive ability and gradually matures during late adolescence (Luna et al., 2015; Calabro et al., 2020), is negatively regulated by glucocorticoids released by the HPA axis and exhibits a projection relationship with the paraventricular nucleus of the hypothalamus (Herman and Cullinan, 1997). Therefore, exposure to METH during adolescence may cause delayed damage to hippocampal maturation by activating the HPA axis, particularly the paraventricular nucleus of the hypothalamus (McKenna and Vertes, 2004; Zhou and Zhu, 2019). These events may explain the damaging effect of METH exposure during adolescence on memory impairment in adulthood, as observed in this study.

Our research confirmed that the decline of learning and memory abilities was accompanied by the changes of synaptic plasticity in the dorsal hippocampus. Here, we first found that the expression levels of *Arc*, *c-fos*, and *Bdnf* were significantly reduced in the dorsal hippocampus of mice suffering from memory impairment in adulthood. As immediate early genes, the rapid and transient response of *Arc* and *c-fos* to synaptic activation is related to long-term potentiation (LTP) maintenance and memory formation (Minatohara et al., 2015). The main role of *Bdnf* is to initiate a signal cascade by binding to tyrosine kinase receptor B and regulating neuronal plasticity that is conducive to cognition (Hogan et al., 2020). The expression levels of these molecules have been widely confirmed to be altered by psychostimulant drugs (Cheng et al., 2015). This suggests that exposure to METH during adolescence leads to the changes in molecules related to neuroplasticity in the dorsal hippocampus in adulthood. We then found that the length of the dorsal hippocampus presynaptic active zone (Figure 4F), an electron-dense network structure in the presynaptic membrane that is important for rapid vesicle fusion and the transmission rate of neurotransmitters in synapses, was reduced in the METH group. Previous studies showed that damage to the presynaptic membrane or a reduction in the length of the presynaptic active zone in the hippocampus could lead to cognitive impairment (Hara et al., 2014; Vasek et al., 2016). Collectively, the available evidence suggests that the synaptic transmission rate in the dorsal hippocampus may be reduced by exposure to METH during adolescence, ultimately contributing to the development of memory impairment (Chen et al., 2020).

Synaptic structural plasticity includes not only changes in the ultrastructure of the synapse but also changes in the number of neurons and glial cells (Allen and Eroglu, 2017). In this study, we found a significantly reduced number of neurons in the CA region of the dorsal hippocampus rather than the DG region in the METH group of mice during adulthood. Studies have shown that METH can induce neuronal damage in the mouse hippocampus *via* peroxidation stress and mitochondrial dysfunction, thus resulting in a decrease in the number of neurons (Park et al., 2017; Veschsanit et al., 2021). During late adolescence, the nervous system begins to mature, including the maturation of the dorsal hippocampus neurons. The cell bodies, synapses, and related neurotransmitter systems of the late maturation region undergo extensive remodeling, thus supporting higher levels of cognitive function (Zeid et al., 2018; Patel et al., 2021). Therefore, exposure to METH during late adolescence may affect these developmental processes, resulting in impaired neuronal maturation, reduced neuronal numbers, and impaired memory in adulthood. Furthermore, the CA region is more susceptible to oxidative stress and pressure than the DG region, thus resulting in structural damage (Ouyang et al., 2007). The activity of pyramidal cells in the CA1–CA3 region was shown to be more active than that of granulosa cells in response to the new environment and spatial coding changes induced by learning (Hainmueller and Bartos, 2018), highlighting the importance of the CA region in new object recognition and spatial memory. A reduction in the number of neurons in the CA region leads to the consequences that are consistent with our behavior performance results in that memory retention in the MWM test, and long-term recognition memory in the NORT, which were found to be impaired in adulthood. Granulosa cells in the DG play a role in initial encoding and the early consolidation of hippocampal memory (Kheirbek et al., 2013). Consistently, we found that in adult mice, learning and short-term memory abilities remained unchanged, since the Y-maze spontaneous alternate test is thought to be a manifestation of short-term memory (Yamada et al., 1996). Combined with the previous findings, our results suggest that exposure to METH during mid-to-late adolescence may cause a reduction of neurons in the CA region which may contribute to the impairment of memory retrieval and long-term recognition ability in adulthood.

In addition, we found that mice in the METH group exhibited an increased synaptic number and PSD thickness, along with a reduced synaptic cleft width, in adulthood. This phenomenon was also reported by the previous studies. For example, blocking neuronal activity with tetrodotoxin or AMPA receptor antagonists can increase the size of synapses and amplify the efficacy of synapses (Turrigiano et al., 1998; Murthy et al., 2001). Intermittent ethanol intake during adolescence was shown to be associated with increased LTP and dendritic spines, but also showed an impaired memory function (Risher et al., 2015; Crews et al., 2016). After several weeks of METH treatment, both the hippocampus and the dorsal striatum showed compensatory effects involving the expression levels of dopamine 1 receptor (D1), dopamine transporter, and tyrosine hydroxylase (Braren et al., 2014). Collectively, we speculate that the increased number of synapses and thickness of PSD, along with the reduced width of the synaptic cleft, in adulthood may arise due to the compensatory effects of homeostatic plasticity mechanisms induced by METH (Davis and Bezprozvanny, 2001).

In general, the morphological and molecular compositions of synapses provide the structural basis for synaptic function (Harris and Weinberg, 2012). Of these, the presynaptic active zone is the site where synaptic vesicles containing neurotransmitters exist (Harris and Weinberg, 2012). Recent studies, involving sections of mature rat hippocampus, reported the recruitment of presynaptic vesicles to nascent areas of pre-existing synapses to convert them into functionally active areas and that this may constitute the initial phase of LTP (Bell et al., 2014). In addition, synaptic vesicles contain cadherins that can modulate AMPAR trafficking, thus contributing to the stabilization of enhanced synaptic efficacy during LTP (Mendez et al., 2010; Harris and Weinberg, 2012). The PSD is the postsynaptic component corresponding to the presynaptic active region, including glutamate receptors directly related to synaptic transmission, as well as a variety of receptors and scaffolds involved in synaptic transmission and plasticity, such as CaMKII and PSD95 (Wheal et al., 1998; Miller et al., 2012). In addition, postsynaptic densities are the heads of dendritic spines, and structural changes in these densities can lead to changes in the synthesis and localization of molecular signaling pathway proteins and cytoskeletal proteins associated with both LTP and LTD (Matsuzaki et al., 2004). The anchoring or internalization of AMPA glutamate receptors produces more functional dendritic spines (LTP) or leads to the contraction of existing dendritic spines (LTD) (Russo et al., 2010). The above-mentioned studies indicated that changes in synaptic structure can lead to the changes in functional plasticity, which ultimately influence behavior. Thus, investigation of how METH exposure during adolescence induces the changes in synaptic structure, and whether it affects functional plasticity and ultimately leads to the mice exhibiting memory impairment in adulthood needs to be further confirmed.

There are some limitations of our research which are need to be considered: (1) the changes in transmission efficiency caused by synaptic structure need to be further verified by synaptic function, (2) the causal relationship between changes in synaptic structural plasticity and memory impairment needs to be confirmed; and (3) there is insufficient evidence to indicate the specific mechanisms underlying memory impairment in mice exposed to METH during adolescence and in particular, whether this involves the activation of the HPA axis and related changes in the neural circuit changes deserves further exploration.

In summary, the exposure of mice to METH during mid-to-late adolescence impaired memory retrieval and long-term recognition memory ability in adulthood. These effects were accompanied by the changes in dorsal hippocampal neuronal and synaptic structure; it is possible that these changes may underlie this delayed impairment, at least in part. These results suggest that by identifying the regulatory mechanisms associated with neuroplasticity changes in this process could provide more evidence to prevent drug abuse in juveniles.

## Data Availability Statement

The original contributions presented in the study are included in the article/supplementary material, further inquiries can be directed to the corresponding author.

## Ethics Statement

The animal study was reviewed and approved by Institutional Animal Care Committee at Xi’an Jiaotong University.

## Author Contributions

TC initiated the project. TC, ML, and LZ designed the experiments. ML and RW carried out the METH exposure and behavior experiments and performed the TEM and IF experiments. ML performed the PCR experiments and wrote the manuscript. RW and HS assisted with data analysis and interpretation of findings. LZ, DM, and HW provided critical revision of the manuscript for intellectual content. All authors critically reviewed the content and approved the final version of the manuscript for publication.

## Conflict of Interest

The authors declare that the research was conducted in the absence of any commercial or financial relationships that could be construed as a potential conflict of interest.

## Publisher’s Note

All claims expressed in this article are solely those of the authors and do not necessarily represent those of their affiliated organizations, or those of the publisher, the editors and the reviewers. Any product that may be evaluated in this article, or claim that may be made by its manufacturer, is not guaranteed or endorsed by the publisher.
